# Xanthoxylin Attenuates Lipopolysaccharide-Induced Lung Injury through Modulation of Akt/HIF-1α/NF-κB and Nrf2 Pathways

**DOI:** 10.3390/ijms25168742

**Published:** 2024-08-10

**Authors:** Fu-Chao Liu, Yuan-Han Yang, Chia-Chih Liao, Hung-Chen Lee

**Affiliations:** 1 Department of Anesthesiology, Chang Gung Memorial Hospital, Linkou Branch, Taoyuan 333, Taiwan; ana5189@cgmh.org.tw (F.-C.L.); m7147@cgmh.org.tw (C.-C.L.); 2 College of Medicine, Chang Gung University, Taoyuan 333, Taiwan; 3 Department of Chinese Acupuncture and Traumatology, Center for Traditional Chinese Medicine, Chang Gung Memorial Hospital, Taoyuan 333, Taiwan; b0105023@cgmh.org.tw

**Keywords:** xanthoxylin, LPS, lung injury, Akt/HIF-1α/NF-κB, Nrf2, ROS

## Abstract

Xanthoxylin, a bioactive phenolic compound extracted from the traditional herbal medicine *Penthorum Chinense Pursh*, is renowned for its anti-inflammatory effects. While previous studies have highlighted the anti-inflammatory and antioxidant properties of Xanthoxylin, its precise mechanisms, particularly concerning immune response and organ protection, remain underexplored. This study aimed to elucidate the effects of Xanthoxylin on inflammation and associated signaling pathways in a mouse model of lipopolysaccharide (LPS)-induced acute lung injury (ALI). ALI was induced via intratracheal administration of LPS, followed by intraperitoneal injections of Xanthoxylin at doses of 1, 2.5, 5, and 10 mg/kg, administered 30 min post-LPS exposure. Lung tissues were harvested for analysis 6 h after LPS challenge. Xanthoxylin treatment significantly mitigated lung tissue damage, pathological alterations, immune cell infiltration, and the production of pro-inflammatory cytokines, including tumor necrosis factor-alpha (TNF-α) and interleukin-6 (IL-6). Additionally, Xanthoxylin modulated the expression of key proteins in the protein kinase B (Akt)/hypoxia-inducible factor 1-alpha (HIF-1α)/nuclear factor-kappa B (NF-κB) signaling pathway, as well as nuclear factor erythroid 2-related factor 2 (Nrf2) and oxidative markers such as superoxide dismutase (SOD) and malondialdehyde (MDA) in the context of LPS-induced injury. This study demonstrates that Xanthoxylin exerts protective and anti-inflammatory effects by down-regulating and inhibiting the Akt/HIF-1α/NF-κB pathways, suggesting its potential as a therapeutic target for the prevention and treatment of ALI or acute respiratory distress syndrome (ARDS).

## 1. Introduction

The lungs are crucial organs, responsible for meeting the body’s basal metabolic demand for oxygen. Acute lung injury (ALI) often leads to inflammatory changes in the lungs, which, if uncontrolled, can escalate to acute respiratory distress syndrome (ARDS)—a severe and widespread manifestation of ALI. ARDS is characterized by decreased lung compliance, severe hypoxemia, and bilateral pulmonary infiltrates, with mortality rates reaching up to 43%, despite a yearly reduction in overall mortality by approximately 1.1% due to advancements in medical care [1,2]. The primary pathological mechanisms of ALI and ARDS involve an acute inflammatory response, marked by the accumulation and activation of inflammatory cells, cytokines, chemokines, and reactive oxygen species (ROS) [3,4,5]. Numerous studies have suggested that anti-inflammatory agents or medications can effectively treat the early stages of ALI and ARDS [6,7,8]. Lipopolysaccharide (LPS), a potent activator of the innate immune system produced by Gram-negative bacteria as part of their outer membrane, is frequently used to induce ALI and inflammation in various animal models [9,10,11].

Alveolar macrophage death significantly influences lung inflammation by affecting other immune cell populations and interacting with alveolar epithelial cells, potentially leading to lung fibrosis [12,13]. Thus, the viability of macrophages is crucial for assessing the effectiveness and safety of treatment strategies. LPS-induced ALI stimulates macrophages to release tumor necrosis factor-alpha (TNF-α) [14]. High concentrations of interleukin-6 (IL-6) serve as predictive markers of poor outcomes in ARDS [15]. Pro-inflammatory cytokines such as TNF-α and IL-6 are biomarkers for predicting mortality and morbidity in ALI and ARDS [3]. Research indicates that inhibiting TNF-α in ALI reduces lung tissue permeability through tight junction regulation [16]. Overexpression of TNF-α and IL-6 not only increases mortality in ALI but also raises the risk of multiple organ dysfunction syndrome [17,18]. Elevated levels of TNF-α and IL-6 have been detected in the bronchoalveolar lavage fluid, lung tissues, and serum of LPS-induced rats [19]. Therefore, TNF-α and IL-6 levels correlate with ARDS severity and reflect the degree of inflammatory response in lung and immune cells.

Nuclear factor erythroid 2-related factor 2 (Nrf2) is a transcription factor that is involved in cellular metabolism and inflammation, regulating cellular responses to oxidative stress through gene expression [20]. Nrf2 governs the expression of antioxidant proteins that protect against oxidative damage triggered by injury and inflammation. Studies have shown that regulating the phosphoinositide 3-kinases (PI3K)/Protein kinase B (Akt)/Nrf2 pathway can reduce lung inflammation [21]. Hypoxia-inducible factor 1-alpha (HIF-1α) mediates adaptive responses to oxidative stress via nuclear translocation and direct mitochondrial targeting [22]. Additionally, the nuclear factor-kappa B (NF-κB) pathway is a pro-inflammatory signaling pathway that regulates cytokines, chemokines, and adhesion molecules, maintaining a balance between pro- and anti-inflammatory responses [23]. Modulating the NF-κB signaling pathway in LPS-induced ALI can reduce TNF-α and IL-6 expression, thereby decreasing mortality risk [17,24].

Xanthoxylin is a bioactive organic compound with notable anti-inflammatory properties [25]. However, there is limited research on the effects and mechanisms of Xanthoxylin in anti-inflammatory contexts. We hypothesize that Xanthoxylin exerts protective effects by reducing lung inflammation through the Akt/HIF-1α/NF-κB and Nrf2 signaling pathways and by regulating pro-inflammatory cytokines such as TNF-α and IL-6. Given the severe mortality risk associated with ARDS and the urgent need for effective anti-inflammatory compounds, we designed experiments to explore the anti-inflammatory pathways and effects of Xanthoxylin using the RAW cell model. Our findings may provide valuable insights for developing ALI treatment medications for both humans and animals.

## 2. Results

### 2.1. Effects of Xanthoxylin Treatment on RAW 264.7 Cells in LPS-Induced Cell Injury

**Figure 1:**
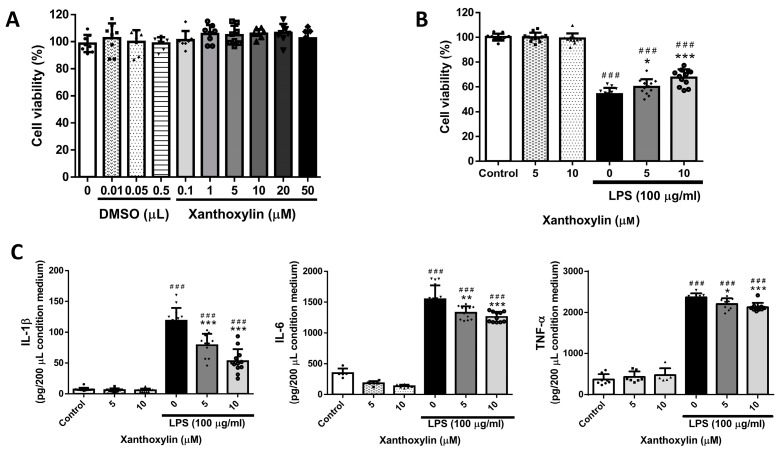
The effect of Xanthoxylin on the viability and pro-inflammatory cytokine levels of RAW 264.7 cells in the presence and absence of LPS. (**A**) RAW 264.7 cells were treated with various concentrations of DMSO (0.01, 0.05, and 0.5 μL) or Xanthoxylin (0.1, 1, 5, 10, 20, and 50 μM) for 24 h. Results are expressed as a percentage relative to the control group and shown as mean ± SD (*n* = 6 per group). (**B**) RAW 264.7 cells were treated with Xanthoxylin (0, 5, and 10 μM) followed by LPS exposure for 48 h to assess cell viability. Results are expressed as a percentage relative to the control group and shown as mean ± SD (*n* = 12 per group). (**C**) Pro-inflammatory cytokines IL-1β, IL-6, and TNF-α levels in the supernatants of RAW 264.7 cells were measured after treatment with Xanthoxylin, followed by LPS exposure. ### *p* < 0.005 vs. control group; * *p* < 0.05, ** *p* < 0.01, *** *p* < 0.005 vs. LPS group.

To investigate the toxicity of Xanthoxylin, we first assessed the viability of RAW 264.7 cells cultured in various solutions and concentrations that would be used in subsequent experiments. As shown in Figure 1A, cell viability and proliferation over 24 h were unaffected by DMSO (0.01–0.5 μL) and Xanthoxylin (0.1–50 μM) solutions. Therefore, the experimental dosages of Xanthoxylin and DMSO had neither a positive nor negative effect on RAW 264.7 cell viability, with no significant differences observed.

To determine whether Xanthoxylin has anti-inflammatory effects, we used LPS to induce inflammation in RAW 264.7 macrophages. As shown in Figure 1B, LPS (100 μg/mL) significantly decreased the viability of RAW 264.7 cells after 48 h. Xanthoxylin did not increase cell proliferation in non-inflammatory conditions but showed significant anti-inflammatory effects in LPS-induced RAW 264.7 cells, particularly at a dosage of 10 μM, increasing cell viability. Additionally, the analysis of culture supernatants revealed that the levels of pro-inflammatory cytokines IL-1β, IL-6, and TNF-α were significantly reduced in the Xanthoxylin treatment groups. These results indicate that Xanthoxylin is non-toxic at concentrations of 0.1–50 μM and enhances cell viability while reducing pro-inflammatory cytokine levels in LPS-induced RAW 264.7 cells.

### 2.2. Effects of Xanthoxylin Treatment on Lung Appearance in LPS-Induced ALI

**Figure 2:**
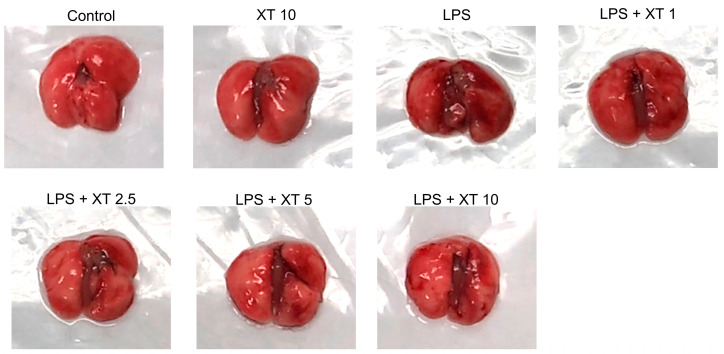
General lung appearance after LPS-induced injury and Xanthoxylin treatment. Mice received an intratracheal LPS challenge followed by intraperitoneal administration of Xanthoxylin (XT, 1, 2.5, 5, and 10 mg/kg) or saline. Lungs were collected 6 h post-LPS challenge for analysis.

An established ALI mouse model was used by exposing mice to intratracheal LPS. After the LPS challenge, mice were sacrificed to evaluate lung histological features and cytokine variations. Lung tissue was harvested 6 h post-LPS challenge. As shown in Figure 2, control groups treated with saline or Xanthoxylin (10 mg/kg) showed no damage. In contrast, the LPS group exhibited extensive edema and hemorrhage. Xanthoxylin administration reduced hemorrhage to varying degrees, with the 10 mg/kg Xanthoxylin group showing the least hemorrhage and the most distinct borders. These results suggest that Xanthoxylin can decrease tissue damage and hemorrhage.

### 2.3. Histological Pattern Changes of Xanthoxylin Treatment on H&E and Inflammatory Cell Infiltration in LPS-Induced ALI

**Figure 3:**
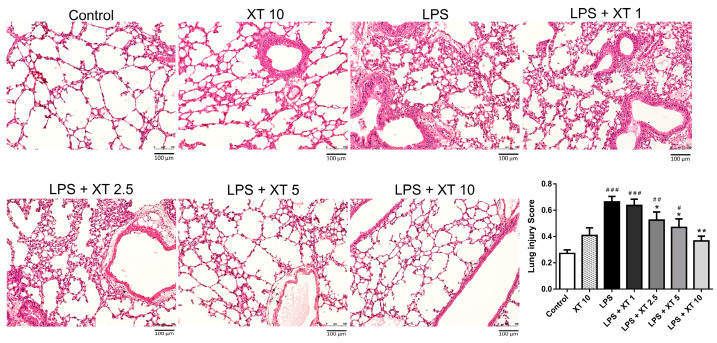
Histological examination of lung tissues stained with H&E after LPS challenge and Xanthoxylin treatment. Mice received LPS intratracheally and were then treated with Xanthoxylin (XT, 1, 2.5, 5, and 10 mg/kg) or saline intraperitoneally. Lungs were harvested 6 h post-LPS challenge for H&E staining. Representative images show ALI and histological changes (100× magnification, scar bar = 100 μm). Quantification of histologic lung injury was analyzed according to American Thoracic Society (ATS) scoring system (*n* = 6 per group). # *p* < 0.05, ## *p* < 0.01, ### *p* < 0.005 vs. control group; * *p* < 0.05, ** *p* < 0.001 vs. LPS group.

H&E staining was used to examine the lung tissue’s microstructure and histological features. Lungs were harvested for H&E staining 6 h after the LPS challenge. As shown in Figure 3, the control and Xanthoxylin (10 mg/kg) control groups exhibited normal lung tissue. The LPS group showed extracellular effusion, lymphocytic cell infiltration, and tissue necrosis. The alveolar structure was severely damaged. Treatment with Xanthoxylin (1, 2.5, 5, and 10 mg/kg) post-LPS challenge significantly reduced pathological damage, with the 10 mg/kg Xanthoxylin group showing well-preserved alveoli and minimal infiltration, effusion, and necrosis. This indicates Xanthoxylin’s ability to mitigate lung tissue inflammation in LPS-induced ALI.

**Figure 4:**
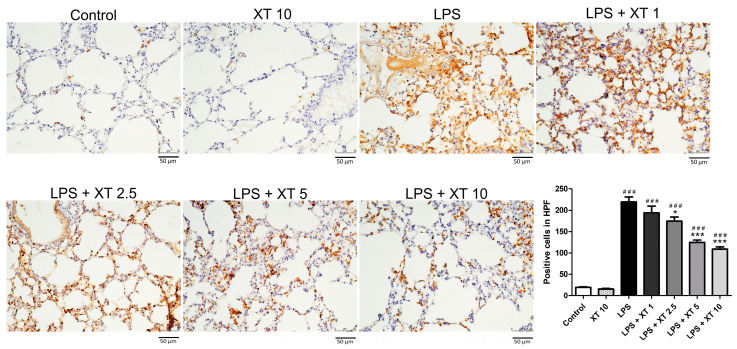
Neutrophil infiltration in lungs following LPS-induced injury and Xanthoxylin treatment. Mice were challenged with LPS intratracheally and treated with Xanthoxylin (XT, 1, 2.5, 5, and 10 mg/kg) or saline intraperitoneally. Lungs were collected 6 h post-LPS challenge and immunostained with Ly6G antibody (200× magnification, scar bar = 50 μm). Quantification of positive cells was analyzed under high power field (HPF). Data are mean ± SD (*n* = 6 per group). ### *p* < 0.005 vs. control group; * *p* < 0.05, *** *p* < 0.005 vs. LPS group.

To assess the neutrophil infiltration and accumulation, lung sections were stained with Ly6G antibody, a granulocyte marker [26]. As shown in Figure 4, the LPS group had a prominent neutrophil presence, while the control and Xanthoxylin (10 mg/kg) groups had minimal staining. Xanthoxylin treatment (1, 2.5, 5, 10 mg/kg) reduced neutrophil infiltration in a dose-dependent manner, with the 10 mg/kg group showing the most significant reduction.

**Figure 5:**
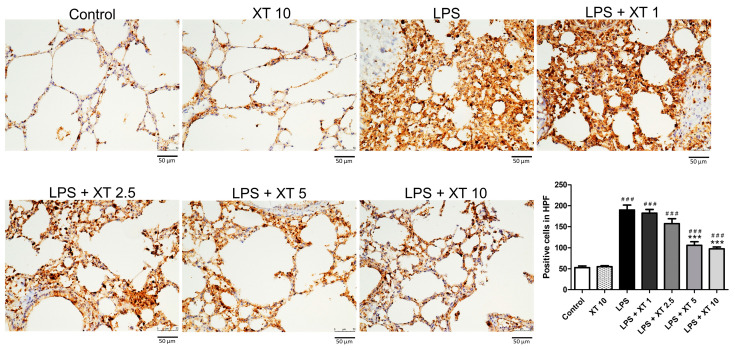
Macrophage infiltration in lungs following LPS-induced injury and Xanthoxylin treatment. Mice received LPS intratracheally and were treated with Xanthoxylin (XT, 1, 2.5, 5, and 10 mg/kg) or saline intraperitoneally. Lungs were harvested 6 h post-LPS challenge and immunostained with Mac-2 antibody (200× magnification, scar bar = 50 μm). Quantification of positive cells was analyzed under HPF. Data are mean ± SD (*n* = 6 per group). ### *p* < 0.005 vs. control group; *** *p* < 0.005 vs. LPS group.

Macrophage infiltration was assessed using Mac-2 antibody staining [2]. As shown in Figure 5, the LPS group had higher macrophage accumulation compared to the control and Xanthoxylin (10 mg/kg) control groups. Xanthoxylin treatment (1, 2.5, 5, 10 mg/kg) reduced macrophage infiltration, with the 10 mg/kg group showing the most significant improvement in alveolar structure.

### 2.4. Effects of Xanthoxylin Treatment on Pulmonic Pro-Inflammatory Cytokines in LPS-Induced ALI

**Figure 6:**
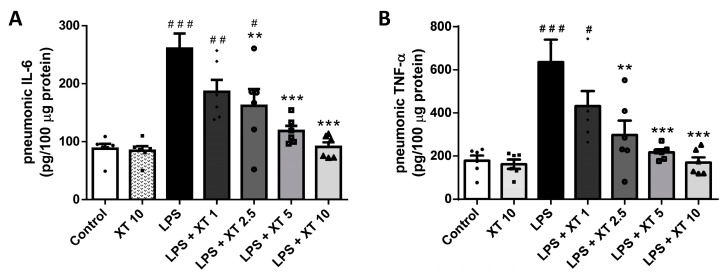
Levels of (**A**) IL-6 and (**B**) TNF-α in lungs after LPS challenge and Xanthoxylin treatment. Mice were given intratracheal LPS challenge followed by Xanthoxylin (XT, 1, 2.5, 5, and 10 mg/kg) or saline intraperitoneally. Lungs were harvested 6 h post-LPS challenge for ELISA. Data are mean ± SD (*n* = 6 per group). # *p* < 0.05, ## *p* < 0.01, ### *p* < 0.005 vs. control group; ** *p* < 0.01, *** *p* < 0.005 vs. LPS group.

TNF-α and IL-6 are key inflammatory cytokines in ALI. To evaluate Xanthoxylin’s effect on these cytokines, we measured their levels using ELISA. As shown in Figure 6, the control and Xanthoxylin (10 mg/kg) control groups had normal TNF-α and IL-6 levels. The LPS-induced ALI group showed significantly elevated cytokine levels. Xanthoxylin treatment (1, 2.5, 5, and 10 mg/kg) post-LPS challenge reduced TNF-α and IL-6 levels in a dose-dependent manner, with the 5 and 10 mg/kg groups approaching normal cytokine levels, indicating Xanthoxylin’s anti-inflammatory effects.

### 2.5. Effects of Xanthoxylin Treatment on Pulmonic Oxidative Stress in LPS-Induced ALI

**Figure 7:**
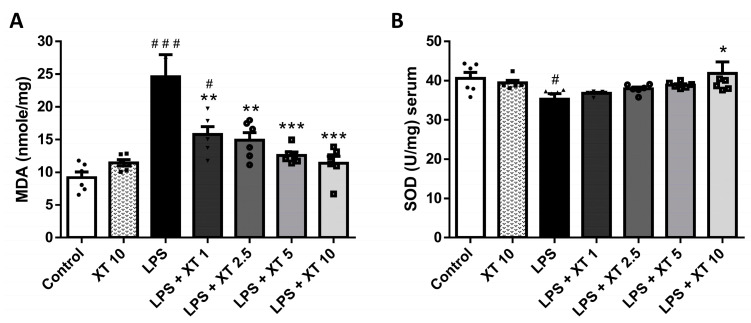
Levels of (**A**) MDA and (**B**) SOD in lungs after LPS challenge and Xanthoxylin treatment. Mice received intratracheal LPS challenge and were treated with Xanthoxylin (XT, 1, 2.5, 5, and 10 mg/kg) or saline intraperitoneally. Lungs were collected 6 h post-LPS challenge for oxidative stress assays. Data are mean ± SD (*n* = 6 per group). # *p* < 0.05, ### *p* < 0.005 vs. control group; * *p* < 0.05, ** *p* < 0.01, *** *p* < 0.005 vs. LPS group.

MDA and SOD levels are indicators of oxidative stress. To investigate Xanthoxylin’s effect on oxidative stress, we quantified the MDA and SOD levels. As shown in Figure 7, the MDA and SOD levels in the Xanthoxylin (10 mg/kg) groups did not differ significantly from controls. The LPS-induced ALI groups showed elevated MDA and decreased SOD levels (*p* < 0.005 and *p* < 0.05, respectively). Xanthoxylin treatment (1, 2.5, 5, 10 mg/kg) significantly inhibited oxidative stress in LPS-induced ALI (*p* < 0.005 for MDA and *p* < 0.05 for SOD), suggesting Xanthoxylin’s role in reducing oxidative stress at 1–10 mg/kg.

### 2.6. Effects of Xanthoxylin Treatment on Pneumonic Akt/HIF-1α/NF-κB and Nrf2 Expression in LPS-Induced ALI

**Figure 8:**
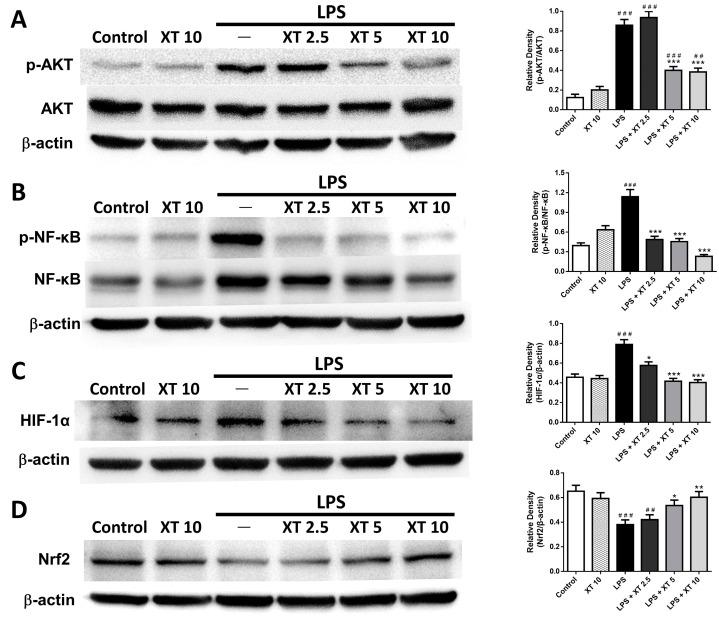
Effects of Xanthoxylin on expression of (**A**) Akt, (**B**) NF-κB, (**C**) HIF-1α, and (**D**) Nrf2 in lungs after LPS challenge. Mice were administered Xanthoxylin (XT, 2.5, 5, and 10 mg/kg) or saline intraperitoneally 30 min post-LPS challenge. Lungs were harvested 6 h later for Western blot analysis. Data are mean ± SD (*n* = 6 per group). ## *p* < 0.01, ### *p* < 0.005 vs. control group; * *p* < 0.05, ** *p* < 0.01, *** *p* < 0.005 vs. LPS group.

Western blot analysis was used to assess the impact of Xanthoxylin on inflammation-associated signaling pathways. As shown in Figure 8A, the total Akt levels did not differ significantly between groups, but phosphorylated Akt increased post-LPS induction and decreased significantly with Xanthoxylin treatment (5 and 10 mg/kg, *p* < 0.005). Figure 8B shows that the NF-κB expression increased significantly post-LPS stimulation and decreased with Xanthoxylin treatment (2.5, 5, 10 mg/kg; *p* < 0.005). Figure 8C indicates a significant increase in HIF-1α expression in the LPS group, which decreased significantly with Xanthoxylin treatment. Figure 8D demonstrates that the Nrf2 expression decreased in the LPS group but increased with Xanthoxylin treatment (5 and 10 mg/kg), reaching statistical significance.

### 2.7. Effects of Xanthoxylin Treatment on Pneumonic Immunostained Nrf2 Antibodies in LPS-Induced ALI

**Figure 9:**
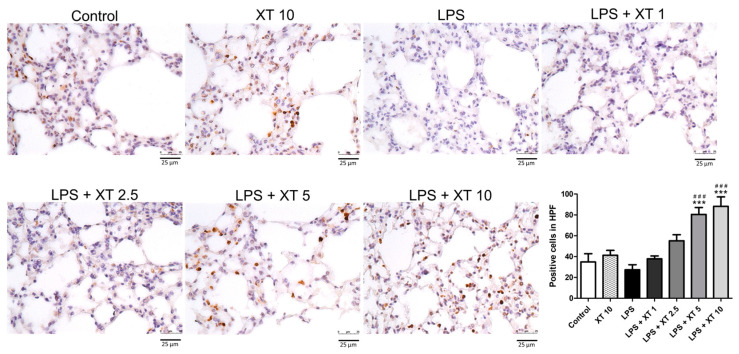
Nrf2 expression in lungs after LPS-induced injury and Xanthoxylin treatment. Mice received intratracheal LPS challenge followed by Xanthoxylin (XT, 1, 2.5, 5, and 10 mg/kg) or saline intraperitoneally. Lungs were collected 6 h post-LPS challenge and immunostained with Nrf2 antibody (400× magnification, scar bar = 25 μm). Quantification of positive cells was analyzed under HPF. Data are mean ± SD (*n* = 6 per group). ### *p* < 0.005 vs. control group; *** *p* < 0.005 vs. LPS group.

Nrf2 is a transcription factor associated with antioxidant proteins. Lung sections stained with Nrf2 antibody showed brown-stained cells indicating Nrf2 expression. As shown in Figure 9, the LPS group had fewer stained cells compared to other groups, while the control and Xanthoxylin (10 mg/kg) control groups had similar Nrf2 expressions. Increasing Xanthoxylin concentrations resulted in increased Nrf2 expression, suggesting that Xanthoxylin stimulates Nrf2 signaling pathways. These results are consistent with those of the Western blot analysis shown in Figure 8.

## 3. Discussion

In this study, ALI was induced in mice using LPS, resulting in significant cellular damage and inflammation. Our investigation focused on the protective effects of Xanthoxylin in these ALI mouse models. Xanthoxylin treatment significantly increased the cell viability in RAW 264.7 cells, reduced histopathological changes, and lowered the levels of pro-inflammatory cytokines, specifically TNF-α and IL-6, thereby mitigating LPS-induced lung tissue damage. Additionally, Xanthoxylin reduced pulmonary neutrophil and macrophage expression in a dose-dependent manner. Furthermore, Xanthoxylin treatment decreased serum MDA levels while increasing SOD activity, indicating a reduction in oxidative stress. Xanthoxylin also suppressed the expression of pneumonic Akt, HIF-1α, and NF-κB proteins, while enhancing Nrf2 expression in LPS-induced ALI lung tissue. These findings suggest that the anti-inflammatory effects of Xanthoxylin may be mediated through the Akt/HIF-1α/NF-κB and Nrf2 signaling pathways.

RAW 264.7 cells, commonly used as a monocyte/macrophage model, play a significant role in LPS-induced ALI in lung tissue [27]. Macrophages, upon exposure to pathogenic organisms or particles such as LPS, can trigger regulated cell death pathways, releasing danger signals into the lung [28]. Research indicates that macrophages and monocytes at inflammation sites are primary sources of TNF-α and IL-6 [29]. Alveolar macrophages produce microvesicles that secrete biologically active TNF-α, initiating ALI, and are key secretors of the pro-inflammatory cytokines found in bronchoalveolar lavage fluid [30,31]. Furthermore, alveolar macrophages can activate neutrophils, which produce a series of pro-inflammatory cytokines, including IL-10 [31].

Our data showed that LPS-induced ALI resulted in reduced cell viability, which was significantly inhibited by Xanthoxylin treatment. Xanthoxylin not only reduced macrophage-regulated cell death but also decreased the release of pro-inflammatory cytokines such as TNF-α and IL-6. Previous research suggests that classical monocytes can enter the airways in response to injury or infection, and elicited macrophages in the interstitium may transition to the airways [32]. TNF-α and IL-6 also stimulate the production and activation of neutrophils, which migrate to inflammation sites to carry out their functions [29]. Our results showed that Xanthoxylin-treated groups had reduced macrophage and neutrophil accumulation, as well as reduced lung tissue damage. The examination of cytology slides stained with H&E revealed that the LPS group exhibited severe lung damage, indicated by significantly higher lung injury scores and compromised alveolar structures. However, Xanthoxylin treatment at 10 mg/kg led to markedly lower lung injury scores, reflecting reduced structural damage to the alveoli.

HIF-1α, a transcription factor that regulates cell metabolism under hypoxia, is known to activate around 60 genes [33]. Under hypoxia, HIF-1α accumulates in the cytoplasm and enters the nucleus to form a stable HIF-1 complex, promoting the transcription of genes that regulate cell metabolism, red blood cell production, cellular energy metabolism, vascular growth, and cell survival [34,35]. In our experiment, the lung tissue showed severe damage after LPS stimulation, and quantitative Western blot analysis showed significant HIF-1α expression post-LPS stimulation. Xanthoxylin treatment mitigated HIF-1α expression, enhancing oxygen conversion efficiency in lung tissue during inflammation.

NF-κB, HIF-1α, and Akt are crucial signaling pathways controlling cellular inflammatory responses [36]. Western blot experiments showed synchronous changes in these proteins. LPS stimulation significantly increased NF-κB, HIF-1α, and Akt levels, which were reduced to near-control levels by the Xanthoxylin intervention. This indicates that Xanthoxylin plays a significant role in regulating these signaling pathways, effectively reducing the inflammatory response and tissue damage caused by LPS-induced ALI.

Nrf2 regulates antioxidant responses and cellular detoxification pathways. Under oxidative stress, toxicity, or inflammation, Nrf2 activates and translocates to the nucleus to regulate genes that are critical for antioxidant enzymes, metabolic enzymes, transporters, and cellular detoxifiers [37]. Nrf2 activation helps cells resist oxidative damage and toxicity, protecting them from disease. In oxidative stress and inflammation, NF-κB and Nrf2 may compete to regulate the same genes [37]. NF-κB typically induces cell death and inflammation, while Nrf2 protects cells from oxidative damage. Therefore, balancing NF-κB and Nrf2 is crucial for disease development [38]. Modulating Nrf2 and NF-κB can reduce inflammation and oxidative damage, aiding in disease treatment [39]. Our experiment indicated that NF-κB and MDA levels increased with LPS stimulation, whereas Nrf2 expression decreased due to NF-κB antagonism. Xanthoxylin treatment effectively increased Nrf2 expression, protecting cells from oxidative damage and toxicity, and increased SOD levels to help clear superoxide radicals from cells.

TNF-α and IL-6 activate the NF-κB pathway, inducing inflammation and cell apoptosis [40]. These cytokines activate IκB protein degradation, releasing NF-κB to enter the nucleus and promote target gene transcription, inducing an inflammatory response [41]. In summary, TNF-α and IL-6 activate the NF-κB pathway, while Nrf2 regulates antioxidant defense, counteracting oxidative stress and inflammatory damage [42]. Regulating NF-κB and Nrf2 through TNF-α and IL-6 is crucial for cellular and tissue health. Targeting these transcription factors may have therapeutic potential for treating inflammatory diseases [43]. Taken together, Xanthoxylin demonstrates potential as a therapeutic agent by modulating the NF-κB and Nrf2 pathways and improving survival rates through a reduction in TNF-α and IL-6 levels in ARDS conditions.

Another important consideration is the source of TNF-α and IL-6. While our results show significant changes in these cytokines, previous studies have indicated that TNF-α and IL-6 primarily originate from macrophages [29]. In this study, the observed tissues are alveolar cells, which differ from macrophages in surface area and volume. This distinction raises the possibility that the measured TNF-α and IL-6 levels may include contributions from aggregated macrophages rather than solely from alveolar cells. This consideration extends to quantitative measurements such as SOD, MDA, and Western blot detection. Despite this, our findings clearly demonstrate that Xanthoxylin exerts significant anti-inflammatory effects by inhibiting the release of TNF-α and IL-6. The observed changes in SOD and MDA levels also suggest that Xanthoxylin may have antioxidant properties, potentially reducing the cellular damage caused by ROS.

**Figure 10:**
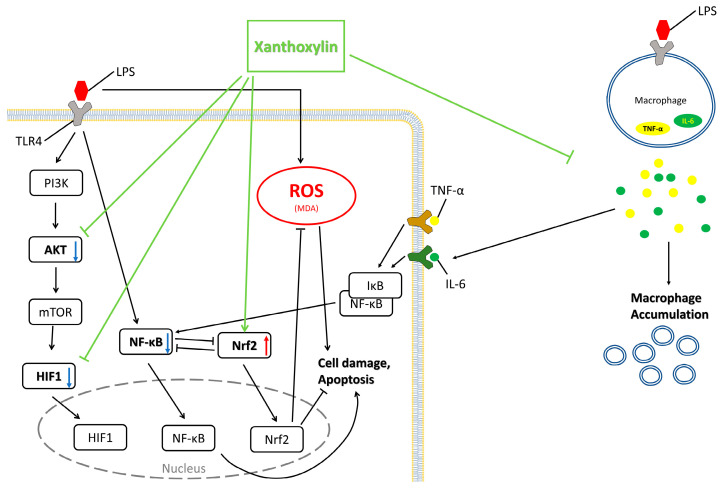
A schematic representation of the involvement of Akt/HIF-1α/NF-κB and Nrf2 signaling pathways in the protective effects of Xanthoxylin against LPS-induced lung injury. Xanthoxylin modulates Akt expression, suppresses HIF-1α/NF-κB signaling, and activates Nrf2, thereby reducing cell damage and oxidative stress. It also inhibits TNF-α and IL-6 release from macrophages.

As shown in Figure 10, LPS binds to Toll-like receptor 4 (TLR4), transmitting the signal to PI3K and subsequently to AKT [44,45]. TLR4 also directly influences NF-κB [45,46]. AKT, a pivotal hub in a signaling network, is commonly associated with inflammatory activation and activates HIF1 via mTOR [47,48]. NF-κB and Nrf2 antagonize each other and regulate apoptosis [49]. HIF1, sensitive to oxidative stress, can be affected by ROS [50]. Macrophages capture LPS, increasing TNF-α and IL-6, which enhance NF-κB signaling through alveolar cell receptors [29,46]. Figure 10 demonstrates the protective effects of Xanthoxylin’s effects on AKT, HIF1, and Nrf2, as well as its inhibition of TNF-α and IL-6.

The recent COVID-19 pandemic has highlighted severe lung damage and symptoms caused by the virus. Severe lung inflammation and infiltration cause dyspnea and hypoxia. COVID-19 infection induces mucus and fluid buildup in the lungs, obstructing airways or alveoli and leading to hypoxia and death. While Xanthoxylin improved LPS-induced ARDS symptoms, further studies are needed to evaluate its effects on virus-induced ARDS. Future research should explore Xanthoxylin as a potential treatment for inflammation, cancer, and immunotherapy.

## 4. Materials and Methods

### 4.1. RAW 264.7 Cells

The murine macrophage cell line RAW 264.7 was maintained in a DMEM medium (Thermo Fisher Scientific, Gibco, Waltham, MA, USA) supplemented with 10% fetal bovine serum (FBS) and antibiotics (100 U/mL penicillin and 100 μg/mL streptomycin), incubated at 37 °C in 5% CO_2_. The cells were sub-cultured every 2–3 days.

### 4.2. Testing Non-Toxic Concentration of RAW 264.7 Cells

RAW 264.7 cells were seeded in 96-well plates at a density of 1 × 10^4^ cells/well and incubated for 24 h at 37 °C in 5% CO_2_. The cells were treated with Xanthoxylin at non-toxic concentrations (0.1, 1, and 5 μM). After 24 h, 10 μL of CCK-8 kit reagent was added to each well and incubated for 2–3 h. Cell viability was measured at 450 nm using a Microplate Reader. Results were expressed as follows: Viability = 100 × (absorbance of treatment/absorbance of control)%.

### 4.3. Cell Culturing Experimental Protocols

RAW 264.7 cells were seeded in 96-well plates at a density of 1 × 10^4^ cells/well overnight. Cells were treated with Xanthoxylin or Dimethyl sulfoxide (DMSO, vehicle control 0.0005–0.001%) in quadruplicate. Viability results were used to assess the cell toxicity of experimental compounds. Cells were incubated with 0, 5, and 10 μM Xanthoxylin for 24 h, followed by treatment with 100 μg/mL LPS or phosphate-buffered saline (PBS, vehicle control). After an additional 48 h, 10 μL of CCK-8 kit reagent was added and incubated for 2–3 h. Cell viability was measured at 450 nm using a Microplate Reader. Results were expressed as follows: Viability = 100 × (absorbance of treatment/absorbance of control)%. Culture supernatants were collected, and pro-inflammatory cytokines were measured using an ELISA kit (Minneapolis, MN, USA).

### 4.4. Animals

C57BL/6 (B6) mice were obtained from BioLASCO Taiwan Co., Ltd. All experimental procedures were approved by the Institutional Animal Care and Use Committee of Chang Gung Memorial Hospital (IACUC No. 2022120102), and the guidelines of the Guide for Care and Use of Laboratory Animals and the Animal Welfare Act were followed. All mice were housed in an environmentally controlled room.

### 4.5. Experimental Protocols and Xanthoxylin Treatment

Mice were housed in an environmentally controlled room and divided into control (saline, Xanthoxylin 10 mg/kg, LPS 2.5 mg/kg) and treatment groups (LPS + Xanthoxylin 1 mg/kg, 2.5 mg/kg, 5 mg/kg, and 10 mg/kg). Mice were anesthetized with ketamine/xylazine (0.15 mL per mouse) and received 2.5 μg/g LPS in 50 μL PBS intratracheally by drop to induce lung injury. After 30 min, mice were treated with different doses of Xanthoxylin as described. Six hours post-LPS challenge, mice were sacrificed, and lung tissues were collected for further analysis.

### 4.6. Histology Examination

Lung tissues were harvested, fixed overnight with 4% paraformaldehyde in PBS, and embedded in paraffin wax. Sections of 4 μm thickness were stained with hematoxylin and eosin (H&E) for histological examination. Histological lung injury score was assessed using the American Thoracic Society (ATS) scoring system [51].

### 4.7. Immunohistochemistry

Tissue sections were blocked for 30 min and incubated with primary antibodies against Ly6G, Mac2, and Nrf2 overnight. After washing, sections were incubated with secondary antibody (biotinylated goat anti-rat or anti-rabbit) for 1 h. Immunostaining was performed using the Millipore IHC Select Kit (MilliporeSigma, Burlington, MA, USA) according to the manufacturer’s protocol.

### 4.8. Measurement of Tissue Cytokine by ELISA

Lung tissues were homogenized on ice, then centrifuged at 12,000× *g* for 10 min at 4 °C. Supernatants were analyzed using the R&D Systems ELISA Kit (Minneapolis, MN, USA) to measure cytokine expression. Primary antibodies were pre-coated on 96-well plates overnight. After blocking for 1 h, supernatants were added and incubated for 2 h at room temperature. Biotinylated detection antibodies were added and incubated for 1 h, followed by incubation with horseradish peroxidase (HRP) substrate for 30 min. The reaction was stopped with 2N H_2_SO_4_, and results were read at 450 nm using a Tecan Infinite 200.

### 4.9. Measurement of the Malondialdehyde (MDA) Levels and Superoxide Dismutase (SOD) Activity

Lung tissue samples were homogenized on ice and centrifuged at 10,000× *g* for 15 min at 4 °C. The supernatant was re-centrifuged at 35,000× *g* for 8 min at 4 °C. To measure lipid peroxidation, the Bioxytech MDA-586 Kit (OxisResearch, Portland, OR, USA) was used, with MDA levels expressed as nmol/g tissue. To assess antioxidant activity, the activity of SOD in serum was determined using a SOD assay kit (Cayman Chemical, Ann Arbor, MI, USA) according to the manufacturer’s protocol. SOD activity was quantified by absorbance at 450 nm and expressed in units (U/mL serum).

### 4.10. Western Blotting

Tissue samples were homogenized in radioimmunoprecipitation assay (RIPA) buffer containing protease inhibitors and sonicated for 15 s. After centrifugation at 12,000× *g* for 10 min at 4 °C, proteins were separated using 10% SDS-PAGE and transferred to polyvinylidene fluoride membranes. Membranes were blocked in 5% fat-free milk for 1 h, washed thrice with Tris-buffer (1% Tween-20), and incubated overnight at 4 °C with primary antibodies against Akt, phospho-Akt, NF-κB, phospho-NF-κB, HIF-1α, and Nrf2. After washing, membranes were incubated with HRP-conjugated secondary antibodies for 1 h at room temperature and visualized using an enhanced chemiluminescence system.

### 4.11. Statistical Analysis

Statistical analysis was performed using Prism Software 6.0. All data were presented as mean ± standard deviation (SD). ANOVA analysis followed by Tukey’s multiple comparison test was used for comparisons.

## 5. Conclusions

Our study demonstrates that Xanthoxylin effectively reduces lung tissue damage and inflammation caused by LPS-induced ALI in mice. Xanthoxylin treatment significantly increased the viability of RAW 264.7 cells, attenuated histopathological changes, and decreased the levels of the pro-inflammatory cytokines TNF-α and IL-6. The protective effects of Xanthoxylin are mediated through the suppression of key inflammatory signaling pathways, including Akt, HIF-1α, and NF-κB and the enhancement of the antioxidant response via Nrf2. This modulation of signaling pathways results in decreased pulmonary neutrophil and macrophage infiltration and reduced overall inflammation and tissue damage. These findings suggest that Xanthoxylin has significant potential as a therapeutic agent for mitigating inflammation and oxidative stress in ALI.

## Data Availability

The data presented in this study are available on request from the corresponding author.
